# P-1573. Methenamine Prophylaxis for Recurrent Urinary Tract Infections

**DOI:** 10.1093/ofid/ofae631.1740

**Published:** 2025-01-29

**Authors:** Hani Salwan AL, Matthew Sims

**Affiliations:** Kaiser Permanente Los Angeles Medical Center, LOS ANGELES, California; William Beaumont University Hospital, Corewell Health, Royal Oak, Michigan

## Abstract

**Background:**

Urinary tract infections (UTIs) are the most common outpatient infection in the United States. Between 50%-60% of women will experience at least one UTI during their life and women over 65 experience UTIs at twice the rate of women overall. Recurrent UTIs are often managed with antimicrobials in clinical practice, which decreases their effectiveness as antimicrobial resistance increases. Methenamine is an antiseptic compound that is converted to formaldehyde under acidic conditions and can prophylactically reduce recurrent UTIs but is seldom used in clinical practice. Our objective is to determine the effectiveness of methenamine for prevention of recurrent UTIs at a large academic medical center in Michigan.

**Methods:**

In this retrospective observational study, 425 patients with recurrent UTIs who were prescribed methenamine for UTI prophylaxis were screened, and 134 who had at least 12 months of pre- and post-methenamine data were included in this study. Patient demographics, medications, UTI history, and kidney function were analyzed.

**Results:**

106 female and 28 male patients with a median age of 80 had 385 UTIs in the year before methenamine prophylaxis and 173 UTIs in the year after methenamine prophylaxis respectively. Methenamine significantly decreased recurrent UTIs across all age groups in both male and female patients. Male patients had an average of 3.4 UTIs in the year prior to methenamine and 1.7 UTIs in the year after methenamine prophylaxis (SD±1.37, p< 0.0001), female patients had an average of 2.7 UTIs in the year prior to methenamine prophylaxis and 1.2 UTIs in the year after methenamine prophylaxis (SD±1.7, p< 0.0001). Methenamine was effective in decreasing recurrent UTIs in patients with chronic kidney disease stages 2, 3a, and 3b. Despite not being indicated for patients with chronic urinary catheters, methenamine decreased UTI recurrence in patients with chronic urinary catheters from 3.5 UTIs in the year before methenamine prophylaxis to 2.1 UTIs in the year after methenamine prophylaxis (SD±1.9, p=0.0004).

**Conclusion:**

Methenamine can serve as a useful prophylactic agent in patients with recurrent UTIs, across all age groups, in patients with stage 2, 3a, and 3b chronic kidney disease, and unexpectedly in patients with chronic urinary catheters.

**Disclosures:**

**Matthew Sims, MD, PhD**, Adaptive Phage Therapeutics: Principal Investigator Clinical Trial|Applied Biocode: Advisor/Consultant|Applied Biocode: Principal Investigator Clinical Trial|Astra Zeneca: Sub-investigator Clinical Trial|Biotest AG: Principal Investigator Clinical Trial|ContraFect: Principal Investigator Clinical Trial|Dompe: Sub-investigator Clinical Trial|Janssen: Principal Investigator Clinical Trial|Leonard-Meron Biosciences: Principal Investigator Clinical Trial|Novozyme: Principal Investigator Clinical Trial|OpGen: Advisor/Consultant|OpGen: Principal Investigator Clinical Trial|Pfizer: Principal Investigator Clinical Trial|Prenosis: Advisor/Consultant|Prenosis: Principal Investigator Clinical Trial|QIAGEN: Principal Investigator Clinical Trial|Seres: Advisor/Consultant|Seres: Principal Investigator Clinical Trial|Venatorx: Advisor/Consultant

